# Recessive *SMC5* Variants in a Family with Near-Tetraploidy/Mosaic Variegated Aneuploidy

**DOI:** 10.3390/diagnostics15233022

**Published:** 2025-11-27

**Authors:** Yongjia Yang, Nian Li, Cheng Liu, Songting Li, Ming Tu, Liu Zhao, Fang Shen, Yu Zheng, Hua Wang, Sha Zhao

**Affiliations:** 1 Department of Medical Genetics, Hunan Children’s Hospital, The Affiliated Children’s Hospital of Xiangya School of Medicine, Central South University, Changsha 410007, China; 2 Department of Medical Genetics, Hunan Children’s Hospital, School of Pediatrics, University of South China, Changsha 410007, China; l1059743275@163.com; 3 Department of Pediatric Healthcare, Hunan Children’s Hospital, The Affiliated Children’s Hospital of Xiangya School of Medicine, Central South University, Changsha 410007, China

**Keywords:** *SMC5*, mosaic variegated aneuploidy, near-tetraploidy, chromosomal instability, Atelis syndrome

## Abstract

**Background/Objectives:** Mosaic variegated aneuploidy (MVA) is a rare chromosomal instability disorder. Biallelic variants in SMC5, a core component of the DNA repair machinery, cause Atelis Syndrome, characterized by severe growth failure and multi-system abnormalities. This study aimed to identify the genetic cause in a patient with MVA and a distinct, milder phenotype. **Methods:** We conducted comprehensive clinical and cytogenetic assessments, chromosomal karyotyping, and trio-based exome sequencing on a proband with hypospadias and chromosomal instability. Identified variants were validated by Sanger sequencing and assessed for pathogenicity using ACMG/AMP guidelines. **Results:** Cytogenetic analysis revealed near-tetraploidy (9.7%) and MVA (46.9%). Exome sequencing identified novel compound heterozygous SMC5 variants, a nonsense c.2221G>T (p.Glu741Ter) and a missense c.3065A>G (p.Asn1022Ser), both predicted to disrupt SMC5/6 complex function. The proband presented with hypospadias and mild developmental delay but lacked the severe neurological, cardiac, or hematological manifestations typical of Atelis Syndrome. Karyotype analysis showed a distinct pattern of chromosomal abnormalities, including a high frequency of marker chromosomes. **Conclusions:** This report expands the genotypic and phenotypic spectrum of SMC5-related disorders, confirming its association with MVA/near-tetraploidy and describing a novel attenuated clinical presentation. The findings highlight distinct cytogenetic patterns potentially differentiating DNA repair-defective MVA from other subtypes.

## 1. Introduction

Mosaic variegated aneuploidy (MVA) is a rare chromosomal instability disorder characterized by tissues containing a mixture of euploid and aneuploid cells with heterogeneous chromosomal gains or losses. Initially described in 1965 as “chaotic chromosomal mosaicism” in siblings with microcephaly and growth retardation [1], MVA has been linked to heightened cancer risk and developmental anomalies [2,3]. To date, pathogenic variants in at least ten genes have been associated with MVA, broadly categorized into two groups based on their mechanistic roles. The first group comprises core components of the spindle assembly checkpoint (SAC) and regulators of centrosome function, including *BUB1B* (MVA1) [4], *BUB1* [5], *MAD1L1* (MVA7) [6], *MAD2L1BP* [7], *TRIP13* (MVA3) [8], *CENATAC* (MVA4) [9], *CEP57* (MVA2) [10], and *CEP192* [11]. Pathogenic variants in these genes disrupt mitotic fidelity, leading to SAC impairment, premature chromosome segregation, and random aneuploidy. The second group involves genes critical for DNA repair and genomic integrity, such as *SMC5* (MVA5) and *SLF2* (MVA6) [12], which primarily perturb DNA replication and repair pathways without directly affecting SAC or centrosomal activity. Notably, while certain SAC gene mutations (e.g., in *BUB1B* or *MAD1L1*) correlate strongly with cancer predisposition, others manifest predominantly as developmental disorders without significant tumorigenesis [6,9,13].

The Structural Maintenance of Chromosomes (SMC) protein complexes—cohesin, condensin, and SMC5–SMC6—are essential for preserving genome stability by regulating chromosome architecture, DNA repair, and segregation [14,15,16]. Among these, the SMC5–SMC6 complex has gained prominence for its vital role in DNA damage response and replication fork protection, with emerging evidence implicating its dysfunction in severe genetic syndromes [17,18,19]. The SMC5–SMC6 complex consists of SMC5 and SMC6 subunits that dimerize into an ATPase module, stabilized by the kleisin subunit NSE4 into a ring-like configuration [20]. This core complex interacts with accessory proteins, including NSE1, NSE2, and NSE3, which provide specialized enzymatic functions: NSE2 acts as a SUMO ligase and NSE1 as a ubiquitin ligase, facilitating post-translational modifications of genome maintenance factors [21,22]. These properties distinguish the SMC5–SMC6 complex from cohesin and condensin, which are primarily involved in chromatin loop extrusion and chromosome condensation [23,24].

Currently, *SMC5* is recognized as the causative gene for Atelis Syndrome 2 (OMIM: 619981). A previous study reported four patients from three families with biallelic *SMC5* variants, presenting with severe microcephaly (down to −7.67 SD), profound short stature (down to −5.68 SD), central nervous system abnormalities, anemia, cardiac defects, and other severe dysmorphic features [12]. The reported variants included p.(Arg425Ter), p.(His990Asp), and p.(Arg372del) [12].

In this study, we describe a novel case of compound heterozygous *SMC5* variants (c.2221G>T, p.(Glu741Ter) and c.3065A>G, p.(Asn1022Ser)) in a patient with MVA/near-tetraploidy. In contrast to previously reported cases—which typically involve severe developmental delay as well as severe neurological, hematological, and cardiac manifestations—our patient presented with hypospadias, mild developmental delay, and no significant neurodevelopmental abnormalities, anemia, or cardiac defects. Cytogenetic analysis revealed a distinctive chromosomal instability profile, including MVA (affecting 46.9% of cells, encompassing both numerical and structural anomalies) and near-tetraploidy (9.7%) in peripheral blood. These findings expand both the phenotypic and genotypic spectrum of *SMC5*-related disorders and provide new mechanistic insights into MVA syndrome pathogenesis.

## 2. Materials and Methods

### 2.1. Study Participants

Three individuals from one family (designated as I-1, I-2, and II-1) were enrolled at Hunan Children’s Hospital. Peripheral blood samples were obtained from each for chromosomal karyotyping and genomic DNA extraction. In addition, archived karyotype slides from ten anonymous pediatric patients (Supplementary Materials Table S1)—each presenting with undiagnosed genetic disorders despite previous karyotyping, exome sequencing, and CNV-seq analyses—were incorporated to evaluate tetraploid metaphases. The study protocol was approved by the Ethics Committee of Hunan Children’s Hospital (Approval No: HCHLL201558, Changsha, Hunan, China) for the index family. Written informed consent was provided by all participants or their legal guardians. The use of archived slides from anonymous cases was exempt from ethics review.

### 2.2. Cytogenetic Analysis

GTG-banding karyotyping was conducted at a resolution of 400–550 bands. Heparinized peripheral blood lymphocytes were cultured in RPMI 1640 medium containing phytohemagglutinin. Following a 72 h incubation period at 37 °C under 5% CO_2_, the cells were harvested through standard procedures involving colcemid arrest and hypotonic KCl treatment. Metaphase spreads were prepared on glass slides and stained with Giemsa-Trypsin-Wright solution. For every sample, two independent cultures were established, and a minimum of 40 metaphase cells were analyzed to confirm karyotype accuracy.

### 2.3. Exome Sequencing and Data Analysis

Exome capture was performed using the iGeneTech T600V1G kit (iGeneTech, Beijing, China), covering a 54 Mb target region. Briefly, 500 ng of genomic DNA meeting quality standards (A260/280 ratio 1.8–2.0) was fragmented into 180–280 bp inserts using a Covaris S220 ultrasonicator (Covaris, Woburn, MA, USA). The fragments were end-repaired, adapter-ligated, and amplified with 8 cycles of PCR. Hybridization was carried out at 65 °C for 24 h, and captured libraries were enriched using magnetic beads. Library quality was assessed using the Agilent Bioanalyzer DNA 2100 Kit (Agilent, Santa Clara, CA, USA) and Qubit 3.0 Fluorometer (Invitrogen, Waltham, MA, USA). Qualified libraries were sequenced on the Salus Pro platform (Salus, Shenzhen, China) with 150 bp paired-end reads. For bioinformatic processing, raw sequencing data were first assessed for quality using FASTQC. Reads were then aligned to the human reference genome (hg38: https://hgdownload.soe.ucsc.edu/goldenPath/hg38/bigZips/hg38.fa.gz) using the Burrows–Wheeler Aligner (BWA) [25]. The resulting SAM files were sorted by coordinate using SortSam [26], and PCR duplicates were marked and removed with MarkDuplicates [27] to improve alignment accuracy and reduce variant calling artifacts. The processed BAM files were re-sorted and indexed using SAMtools to enable efficient data access. Variant calling was performed with the Genome Analysis Toolkit (GATK) [28], a widely recognized framework for identifying SNPs and indels, which generated variant calls in VCF format.

### 2.4. Variant Confirmation and Interpretation

Variants in *SMC5* were confirmed via Sanger sequencing using previously described methods [29]. The primers used were as follows: for c.2221G>T p.(Glu741Ter), forward: 5′-actctccccactttagctcc-3′ and reverse: 5′-gcaactaagcaagctgaaactg-3′ (product size 499 bp); for c.3065A>G p.(Asn1022Ser), forward: 5′-aactcttggggaagcctact-3′ and reverse: 5′-ccgttcattgattgggtcca-3′ (product size 589 bp). All variants were classified according to the guidelines issued by the American College of Medical Genetics and Genomics (ACMG) and the Association for Molecular Pathology (AMP) [30].

### 2.5. Evolutionary Conservation Analysis & Protein Structure Prediction

Evolutionary conservation of SMC5 was analyzed through multiple sequence alignment of vertebrate orthologs from NCBI Protein database. Homology modeling was used to predict the structure using SWISS-MODEL.

## 3. Results

### 3.1. Clinical Presentation and Initial Karyotype Analysis Revealing Near-Tetraploidy/MVA

**Figure 1:**
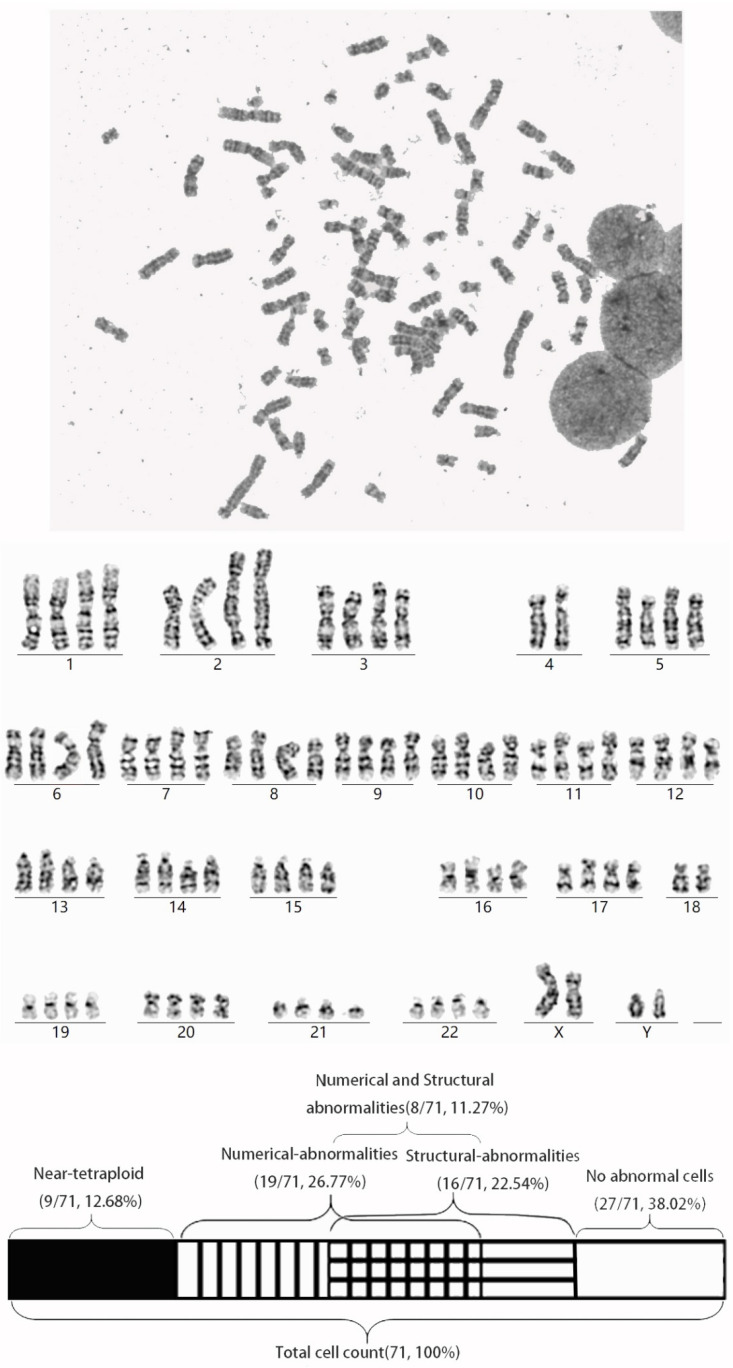
Karyotype analysis of the proband reveals a high frequency of complex chromosomal abnormalities. (**Upper panel**) A representative near-tetraploid karyogram (88,XXYY) with structural anomalies, including losses of chromosomes 4 and 18, and a pseudodicentric derivative chromosome: 88,XXYY,−4,−4,−18,−18,psu dic(2)t(4;2)(q34;q21), 21ps-×2. (**Lower panel**) Summary of cytogenetic findings. Among 71 metaphase cells analyzed, 44 (62.0%) exhibited chromosomal abnormalities. These included cells with numerical defects only (26.8%), structural defects only (22.5%), and a notable subset (11.3%) showing both numerical and structural aberrations concurrently.

A male infant presented to our clinic with hypospadia. At 1 year and 8 months of age, his height was 79.5 cm (between −2SD and −1SD), weight was 9.7 kg (approaching −2SD), and head circumference was 46.3 cm (between −2SD and −1SD). Physical examination confirmed hypospadias with bilaterally palpable testes. Ultrasonography revealed normal renal architecture and testicular sizes (left: 15 × 7 × 6 mm; right: 15 × 7 × 7 mm). The patient demonstrated age-appropriate neurodevelopment without dysmorphic features and had an unremarkable birth history. At 4 years and 2 months of age, the anthropometric parameters of the proband (II:1)—including height, weight, and head circumference—had all returned to the normal range (25th–50th percentiles). Parental consanguinity was denied; the parents were 29 and 28 years old at the time of delivery. Standard cytogenetic analysis was performed. A systematic comparison of the proband’s clinical features with other reported cases of *SMC5*-related Atelis syndrome is provided in Table 1, highlighting the distinctly milder phenotypic presentation. Karyotyping of 71 metaphase cells revealed abnormal ploidy in 36 cells (50.7%), specifically, 9 cells (12.7%) showed near-tetraploidy and 27 cells (38.0%) exhibited MVA (Figure 1; Supplementary Materials Table S2). Both parents exhibited normal karyotypes.

### 3.2. Identification of SMC5 Variants

**Figure 2:**
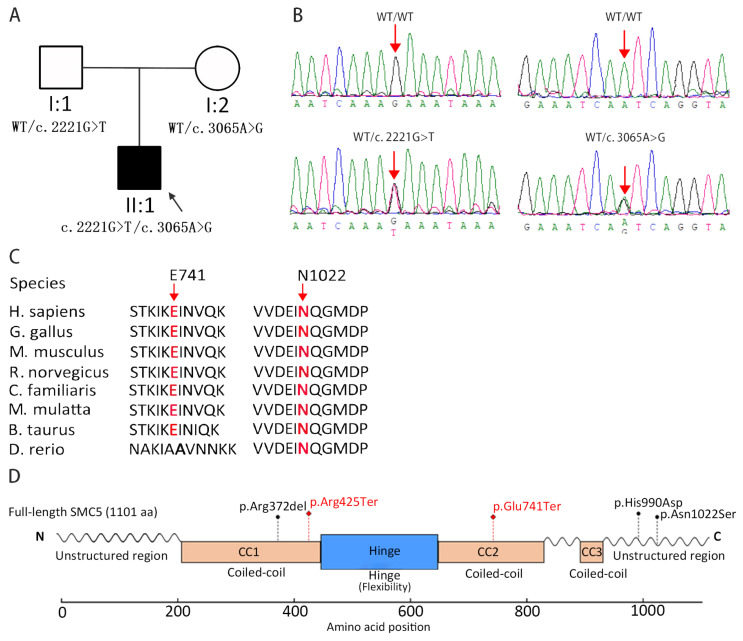
Schematic representation of *SMC5* variants identified in patients with MVA. (**A**) Compound heterozygous variants in *SMC5* detected by exome sequencing in the present pedigree. Note: WT = wild type. (**B**) Validation of the compound heterozygous *SMC5* variants by Sanger sequencing. (**C**) Both *SMC5* variants identified in this study are located in conserved regions of the protein amino acid sequence. Note: E741 corresponds to the c.2221G>T, p.(Glu741Ter) variant; N1022 corresponds to the c.3065A>G, p.(Asn1022Ser) variant. (**D**) Locations of the two *SMC5* variants identified in this study (highlighted in red), compared with the previously reported biallelic *SMC5* variants associated with MVA (shown in black) [12]. Note: Both p.(Glu741Ter) and p.(Arg425Ter) are premature termination variants, which are predicted to result in truncated protein products.

Exome sequencing was performed on the proband (II-1) and both parents (I-1 and I-2) (Figure 2A). Given that MVA syndrome is a rare autosomal recessive disorder [4,5,6,7,8,9,10,11,12], we prioritized rare biallelic variants in known MVA-associated genes [4,5,6,7,8,9,10,11,12] in the proband using a stepwise filtering strategy (Supplementary Materials Figure S1). Two compound heterozygous variants in SMC5 (NM_015110.4: c.2221G>T, p.(Glu741Ter) and c.3065A>G, p.(Asn1022Ser)) were identified as the only variants satisfying all filtering criteria (Figure 2A) (Table 2). Segregation analysis by Sanger sequencing confirmed that these variants co-segregated with the phenotype within the family (Figure 2B). No other rare or pathogenic variants were detected in known MVA-related genes. According to the ACMG/AMP guidelines, the c.2221G>T (p.Glu741Ter) variant was classified as Pathogenic (PVS1, PM2, PP4), and the c.3065A>G (p.Asn1022Ser) variant was assessed as Likely Pathogenic (PM2, PP3, PP4, PM3).

### 3.3. Evolutionary Conservation and Protein Structural Analysis

SMC5 is a 1101-residue protein containing three coiled-coil (CC) domains in its central region. The p.(Asn1022Ser) variant (nearing to a known pathogenic variant of *SMC5*, i.e., p.(His990Asp) [12]) is situated in the C-terminal region; the Asn1022 residue exhibits high evolutionary conservation across species (Figure 2C).

**Figure 3:**
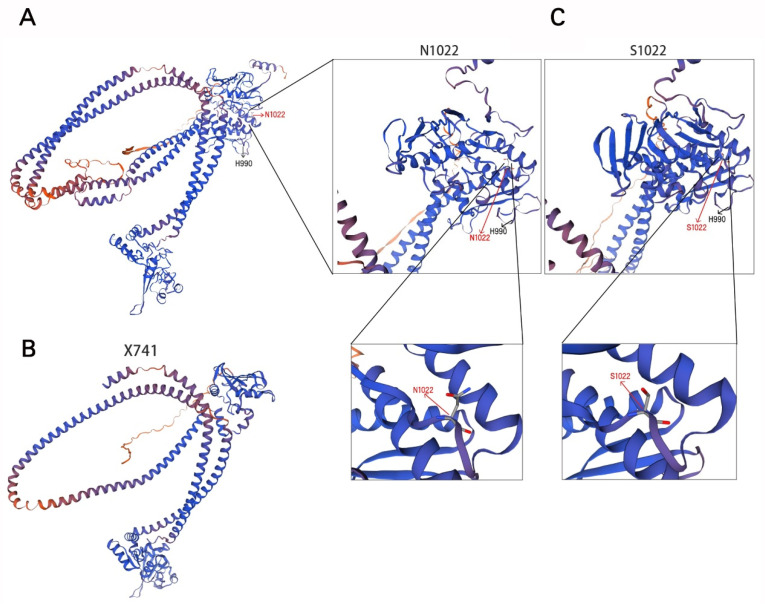
Structural implications of *SMC5* variants. (**A**,**B**) Structural model of the SMC5 (simplified representation), highlighting the C-terminal domain of SMC5 that is truncated by the p.Glu741Ter variant. This region is essential for interaction with NSE4 and complex stability. (**C**) Close-up view of the wild-type (left, Asn1022) and mutant (right, Ser1022) residues within the C-terminal region of SMC5. Substitution of asparagine by serine disrupts potential hydrogen-bonding interactions (Red arrows) due to differences in side-chain length and chemistry, thereby compromising structural integrity and partner binding. Note: The known pathogenic variant p.(His990Asp) position, which corresponds to H990, is denoted by black arrows.

In contrast, the c.2221G>T (p.Glu741Ter) variant introduces a premature termination codon that is predicted to express a truncated protein (Figure 2D) or to trigger nonsense-mediated mRNA decay. C-terminal region of SMC5 is essential for interaction with non-SMC elements (NSEs), particularly NSE4/Kleisin, which plays a critical role in maintaining the structural integrity and ATPase function of the SMC5/6 holo-complex [31,32]. Truncation of the C-terminal (Figure 3A,B) is predicted to prevent proper integration of SMC5 into the complex, leading to defective assembly, reduced structural stability, and potential haploinsufficiency or complete loss of function.

The missense variant (p.(Asn1022Ser)) results in the substitution of asparagine by serine at residue 1022, which lies within the evolutionarily conserved C-terminal tail of SMC5. Structural analyses indicate that this region facilitates specific electrostatic and hydrophobic interactions with NSE4, thereby stabilizing the overall architecture of the SMC5/6 complex [32,33]. Asparagine at this position is likely involved in a hydrogen-bonding network critical for interface stability. Its replacement with serine—which has a shorter side chain and altered hydrogen-bonding capacity (Figure 3C,D)—is expected to disrupt these interactions, leading to impaired complex stability, conformational alterations, and functional deficiency.

### 3.4. Further Analysis of Chromosomal Karyotyping

To confirm the presence of near-tetraploidy/MVA in the individual with compound heterozygous variants in SMC5, we performed a second round of karyotype analysis on peripheral blood samples from the proband (II:1) at the age of 4 years and 2 months. A total of 464 cells were analyzed, revealing 43 near-tetraploid cells (9.3%) and 224 cells with MVA (48.3%), including both numerical and structural chromosomal abnormalities (Supplementary Materials Table S3).

Combining results from two independent karyotypic analyses (a total of 535 cells), we identified 52 near-tetraploid cells (9.7%) and 251 MVA cells (46.9%).

**Figure 4:**
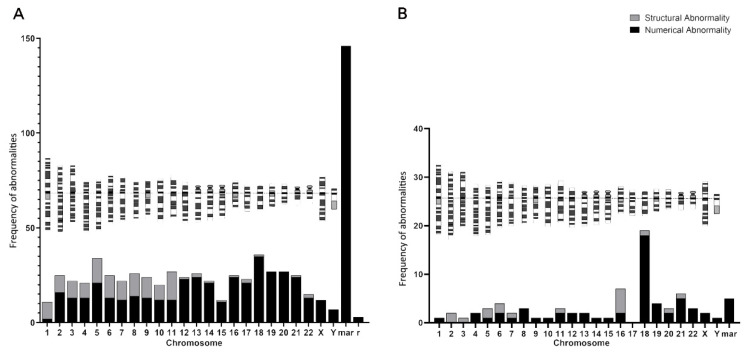
Distribution of chromosomal abnormalities in patient-derived cells. (**A**) Frequency of numerical and structural abnormalities per chromosome in cells (n = 251/483 abnormal cells) from an individual with compound heterozygous *SMC5* variants. Note the markedly higher frequency of abnormalities, particularly structural aberrations on specific autosomes (e.g., chromosome 8) and the high occurrence of marker chromosomes (mar). (**B**) For comparison, the frequency of abnormalities in cells (n = 72/232 abnormal cells) from two individuals with *CEP192* variants (data from Supplementary Materials Table S3). The overall burden of abnormalities is lower, and the distribution pattern is distinct, with a notable prevalence of numerical abnormalities on chromosome 18.

Classification of all MVA karyotypes by chromosome revealed that longer chromosomes exhibited fewer numerical abnormalities but a higher frequency of structural abnormalities (Figure 4A). The combined frequency of numerical and structural abnormalities showed no significant bias across chromosomes (Figure 4A). Among all aberrations, marker chromosomes were the most prevalent (Figure 4A). For comparison, we analyzed karyotypic data (Supplementary Materials Table S4) from individuals with biallelic variants in *CEP192*—a gene previously implicated in spindle defects, leading to MVA and tetraploidy [11]. In contrast to the *SMC5* case, the *CEP192* group showed no correlation between chromosome length and the frequency of either numerical or structural abnormalities (Figure 4B). Furthermore, marker chromosomes were significantly less frequent in *CEP192* individuals than in the *SMC5* case.

The emergence of tetraploid or near-tetraploid cells in both *SMC5*- and *CEP192*-related variants represents a novel finding. To evaluate the specificity of this observation, we analyzed 100 metaphase cells from each of 10 children with congenital structural abnormalities. Tetraploid cells were rare—observed in only one cell from a single individual (0.1% per sample, on average)—indicating that the high frequency of tetraploidy (9.7%) in the SMC5 proband reflects a bona fide chromosomal anomaly rather than stochastic background occurrence.

### 3.5. Chromosomes with Unusual Structural Abnormalities

**Figure 5:**
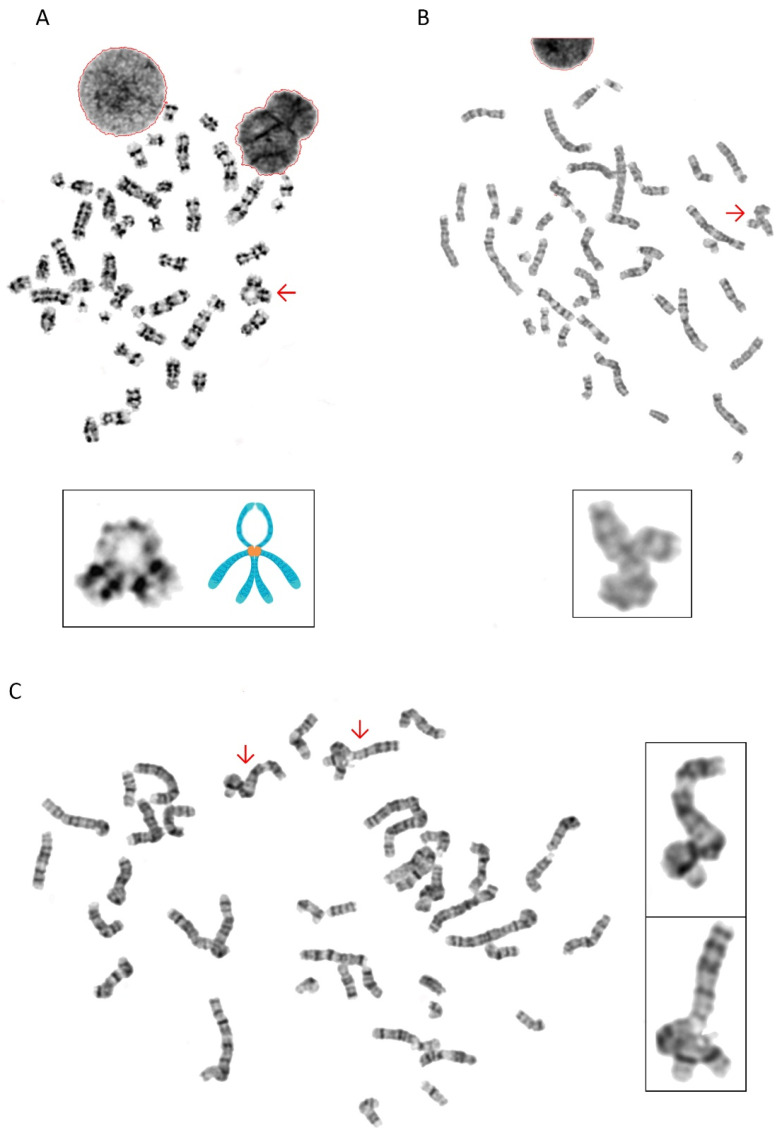
Unusual chromosomal configurations in patient-derived cells. (**A**) A distinctive arched-ring chromosome, likely formed by two sets of the long arms of chromosome 16 (4n) connected at the centromere, with two short arms (2n) composing the ring structure. (**B**,**C**) Examples of misattachments involving three or more chromosomal fragments. These previously unrecognized structural anomalies suggest aberrant repair mechanisms and chromosomal missegregation in the context of *SMC5* deficiency. The red arrows point to abnormally structured chromosomes.

During karyotypic analysis, we occasionally observed previously unrecognized chromosomal structures with unusual configurations: (1) a distinctive arched-ring chromosome, likely formed by two sets of chromosomes with 16 long arms (4n) connected at the centromere, with two short arms (2n) composing the ring structure (Figure 5A), and (2) misattachments involving three or more chromosomal fragments (Figure 5B,C).

## 4. Discussion

The RAD18–SLF1/2–SMC5/6 pathway constitutes a linear protein recruitment cascade that is essential for the cellular response to DNA replication stress [34]. Through ubiquitin signaling and specific protein–protein interactions, this pathway mediates the precise targeting of the SMC5/6 complex—a central guardian of genome integrity—to sites of DNA damage [34,35]. By promoting the resolution of stalled replication forks and preventing the accumulation of DNA replication errors, the RAD18–SLF1/2–SMC5/6 axis serves as a fundamental mechanism for maintaining genomic stability in vertebrate cells [34,35]. As a core component of this pathway, *SMC5* has recently been implicated in the etiology of MVA syndrome, specifically Atelis syndrome [12]. In this study, we identified two novel *SMC5* variants that are predicted from a structural standpoint to severely compromise the assembly and function of the SMC5/6 complex. These findings are consistent with the clinical manifestations observed in the patient, including reduced height, weight, and head circumference, as well as chromosomal aneuploidy [12]. The p.(Glu741Ter) nonsense variant is expected to result in a complete loss of function of the affected allele. The p.(Asn1022Ser) missense variant, although not truncating, affects a critical interface required for the interaction between SMC5 and NSE4/Kleisin [32,33]. In summary, the p.(Glu741Ter) and p.(Asn1022Ser) variants impair SMC5/6 complex function through distinct molecular mechanisms—protein truncation and disruption of a key binding interface, respectively—thereby providing a solid molecular genetic basis for the patient’s clinical phenotype. These findings not only expand the spectrum of pathogenic mutations in *SMC5* but also underscore the central role of the SMC5/6 complex in maintaining genome stability.

It must be noted that the patient described in our study exhibited hypospadias and mild deficits in height, weight, and head circumference at 1 year and 8 months of age—interestingly, these growth parameters had normalized by the age of 4 years and 2 months. Furthermore, the patient presented without significant neurodevelopmental abnormalities, anemia, or cardiac defects. This clinical presentation differs notably from previously reported cases, which often include severe developmental delay and pronounced neurological, hematological, and cardiac manifestations [12]. This divergence may be attributed to differences in the location and type of mutations: both p.(Glu741Ter) and p.(Asn1022Ser) identified here are novel variants. While p.(Glu741Ter) is predicted to cause haploinsufficiency or a complete loss of function—similar to the previously reported truncating variant p.(Arg425Ter)—the p.(Asn1022Ser) variant may lead to a partial impairment of SMC5 function. In contrast, the p.(His990Asp) variant has been associated with severe symptoms in all three reported patients [12], suggesting that it may disrupt the full functionality of SMC5. We cannot exclude the possibility that additional clinical features may emerge in our patient with advancing age, warranting long-term follow-up.

Another noteworthy aspect of this study lies in the chromosomal karyotype findings. First, we detected near-tetraploid cells in the individual with biallelic *SMC5* variants—a phenomenon not previously reported in other *SMC5*-related cases [12]. Interestingly, a similar tetraploid cell population was also observed in a recently reported family with MVA syndrome due to biallelic *CEP193* mutations [11], suggesting that tetraploidization may represent a common feature among MVA syndrome patients, although this observation requires further validation in larger cohorts. Second, we attempted to characterize and compare the distribution of chromosomal abnormalities in our *SMC5*-related case and in available *CEP192*-related cases. Preliminary observations indicate potential divergences: the *SMC5* case exhibited a higher incidence of marker chromosomes, while *CEP192* cases showed relatively fewer. Among autosomal abnormalities, chromosome 18 was most frequently involved in *CEP192* cases, whereas chromosome 8 was most commonly affected in the *SMC5* case. Additionally, in the *SMC5* patient, longer chromosomes appeared to harbor fewer numerical abnormalities but more structural alterations. These distinct patterns may eventually aid in differentiating between SAC-defective MVA (e.g., *CEP192*-related) and DNA repair-defective MVA (e.g., *SMC5*-related), though additional data from more cases are needed to substantiate this possibility.

Our study has several limitations that should be considered when interpreting the results. First, the findings are derived from a single proband and a small control cohort (n = 10), which limits the generalizability of our conclusions. Although the control individuals were not precisely age-matched (ages ranged from 3.8 to 10.7 years; Supplementary Materials Table S1), their ages overlapped with that of the proband (4 years and 2 months at the time of the second karyotype analysis). The extremely low frequency of tetraploid cells in controls (0.1% on average) compared to the proband (9.7%) suggests that the observed difference is unlikely to be attributable solely to age variation. The potential for selection bias also exists, as our cohort may not fully represent the broader phenotypic spectrum of MVA syndrome, particularly including individuals with milder forms who do not present to clinical attention. Second, while we identified the compound heterozygous SMC5 variants [p.(Glu741Ter) and p.(Asn1022Ser)] through whole-exome sequencing, their functional impact was assessed primarily through bioinformatic predictions. Future studies employing in vitro or in vivo functional assays are necessary to definitively confirm their pathogenic mechanisms. In conclusion, our study represents the second report of biallelic *SMC5* variants causing an MVA-related disorder. These findings expand the mutational spectrum of *SMC5* and reinforce its critical role in chromosomal integrity.

## Figures and Tables

**Table 1:** Comparative Clinical Features of Patients with Biallelic *SMC5* Variants.

Clinical Feature	P7 [12]	P8 [12]	P9-1 [12]	P9-2 [12]	Present Patient
Demographics & Genetics					
Origin	Spain	US	US	US	China
SMC5 Variants	c.1110_1112del; p.(Arg372del)/	c.2970C>G; p.(His990Asp)/Homozygous	c.2970C>G; p.(His990Asp)/Homozygous	c.2970C>G; p.(His990Asp)/Homozygous	c.2221G>T; p.(Glu741Ter)/
	c.1273C>T; p.(Arg425Ter)				c.3065A>G; p.(Asn1022Ser)
Growth & Development					
Prenatal/Birth Parameters	Severe IUGR	IUGR, SGA	IUGR	IUGR, Premature	Within normal limits
	(−4.25 SD)	(−1.45 SD)	(−2.87 SD)	(−1.56 SD)	
Postnatal Short Stature	+ (Severe, −5.68 SD)	+ (−2.94 SD)	+ (−5.87 SD)	+ (−4.65 SD)	**-** (Mild delay, normalized by age 4)
Microcephaly	+ (Severe, −7.67 SD)	+ (−4.65 SD)	+ (−4.88 SD)	+ (−6.93 SD)	**-**
Neurological & Development					
Global Developmental Delay/Intellectual Disability	+ (Learning difficulties)	- (Motor delay due to blindness)	-	- (Mild resolved motor delay)	+ (Mild, resolved)
Seizures	Not reported	+	Not reported	Not reported	**-**
Behavioral Anomalies (e.g., Anxiety)	Not reported	+	+	+	**-**
Craniofacial Features					
Dysmorphic Facies	+ (Long face, beaked nose, epicanthal folds)	+ (Triangular face, frontal bossing, micrognathia)	+ (Triangular face, frontal bossing)	+ (Triangular face, micrognathia, downslanting palpebral fissures)	Absent
Systemic Involvement					
Cardiac Abnormalities	+ (PDA, Pulmonary stenosis)	+ (Innocent murmur)	+ (Innocent murmur)	+ (Mild pulmonary stenosis, PFO/ASD)	**-**
Ocular Abnormalities	Not reported	+ (Microphthalmia, cataracts, retinal detachment)	Not reported	+ (Persistent fetal vasculature)	**-**
Hematological/Immunological Abnormalities	Not assessed	+ (Lymphopenia, eosinophilia)	+ (Thrombocytopenia, lymphopenia)	+ (Anemia, neutropenia, MDS)	**-**
Skeletal Anomalies	+ (High palate)	-	+ (Clinodactyly, pes planus)	+ (Clinodactyly, brachydactyly, kyphosis)	**-**
Endocrine Anomalies	+ (Hyperinsulinism)	+ (Elevated TSH)	+ (Elevated TSH)	+ (Elevated TSH, premature adrenarche)	**-**
Other	Hypospadias	Sacral dimple, elevated LFTs	Sacral dimple, elevated LFTs	Sacral dimple, pilonidal cyst, elevated LFTs	Hypospadias
Laboratory & Cytogenetic Findings					
Mosaic Variegated Aneuploidy (MVA)	+	+	+	+	**+**
Near-Tetraploidy	Not reported	Not reported	Not reported	Not reported	**+**
Summary: Features Absent in Present Patient *	-	-	-	-	10/13 (76.9%)

Abbreviations: +, present; -, absent/not observed; IUGR, Intrauterine Growth Restriction; SGA, Small for Gestational Age; PDA, Patent Ductus Arteriosus; PFO, Patent Foramen Ovale; ASD, Atrial Septal Defect; MDS, Myelodysplastic Syndrome; LFTs, Liver Function Tests; TSH, Thyroid-Stimulating Hormone. * The summary row quantifies the phenotypic divergence by calculating the proportion of core features typically associated with classical Atelis Syndrome (based on the 4 previously reported patients, P7-P9-2) that are absent in the present patient. The 13 features assessed for this calculation are Postnatal Short Stature, Microcephaly, Global Developmental Delay/Intellectual Disability, Seizures, Behavioral Anomalies, Dysmorphic Facies, Cardiac Abnormalities, Ocular Abnormalities, Hematological/Immunological Abnormalities, Skeletal Anomalies, Endocrine Anomalies, Mosaic Variegated Aneuploidy (MVA), and Near-Tetraploidy.

**Table 2:** Detailed characterization of the compound heterozygous variants in the *SMC5* identified in the proband. ^a^: according to gnomAD v4.1.0. -: indicates that the software cannot predict protein truncating variants.

Item	Variant 1	Variant 2
Genomic Coordinate (GRCh37)	chr9: 72938469	chr9: 72965205
Transcript	NM_015110.3	NM_015110.3
cDNA Change	c.2221G>T	c.3065A>G
Protein Change	p.(Glu741Ter)	p.(Asn1022Ser)
Variant Type	Missense	Missense
Zygosity	Heterozygous	Heterozygous
Variant Allele Fraction (VAF)	48%	53%
Population Frequency (gnomAD) ^a^	Not found	0.000006821
In Silico Prediction	CADD: 39 (Damaging)	CADD: 27.1 (Damaging)
	SIFT: -	SIFT: Deleterious
	PolyPhen-2: -	PolyPhen-2: 1
ACMG/AMP Classification	Pathogenic (PVS1, PM2, PP4)	Likely Pathogenic (PM2, PP3, PP4, PM3)
Origin	Paternal	Maternal

Note: “-“ indicates that the software cannot predict protein truncating variants.

## Data Availability

The datasets used and/or analyzed during the current study will be made available from the corresponding authors upon reasonable request.

## Supplementary Materials

The following supporting information can be downloaded at https://www.mdpi.com/article/10.3390/diagnostics15233022/s1, Figure S1: Workflow of variant filtering and analysis; Table S1: Demographic Clinical Characteristics of the Study Participants (as control); Table S2: Results of the Initial Karyotype Analysis of the Proband with SMC5 biallelic variants; Table S3: Results of the Second Karyotype Analysis of the Proband with SMC5 biallelic variants; Table S4: Results of Karyotype Analysis of cases with CEP192 biallelic variants (Excluding Tetraploid Cells, 72 Abnormal Cells Observed).

## References

[1] Kajii T., Ikeuchi T., Yang Z.-Q., Nakamura Y., Tsuji Y., Yokomori K., Kawamura M., Fukuda S., Horita S., Asamoto A. (2001). Cancer-prone syndrome of mosaic variegated aneuploidy and total premature chromatid separation: Report of five infants. Am. J. Med. Genet..

[2] Jacquemont S., Bocéno M., Rival J.M., Méchinaud F., David A. (2002). High risk of malignancy in mosaic variegated aneuploidy syndrome. Am. J. Med. Genet..

[3] Warburton D., Anyane-Yeboa K., Taterka P., Yu C.Y., Olsen D. (1991). Mosaic variegated aneuploidy with microcephaly: A new human mitotic mutant?. Ann. Genet..

[4] Hanks S., Coleman K., Reid S., Plaja A., Firth H., FitzPatrick D., Kidd A., Méhes K., Nash R., Robin N. (2004). Constitutional aneuploidy and cancer predisposition caused by biallelic mutations in *BUB1B*. Nat. Genet..

[5] de Voer R.M., van Kessel A.G., Weren R.D.A., Ligtenberg M.J.L., Smeets D., Fu L., Vreede L., Kamping E.J., Verwiel E.T.P., Hahn M.-M. (2013). Germline mutations in the spindle assembly checkpoint genes *BUB1* and *BUB3* are risk factors for colorectal cancer. Gastroenterology.

[6] Villarroya-Beltri C., Osorio A., Torres-Ruiz R., Gómez-Sánchez D., Trakala M., Sánchez-Belmonte A., Mercadillo F., Hurtado B., Pitarch B., Hernández-Núñez A. (2022). Biallelic germline mutations in *MAD1L1* induce a syndrome of aneuploidy with high tumor susceptibility. Sci Adv..

[7] Abdel-Salam G.M.H., Hellmuth S., Gradhand E., Käseberg S., Winter J., Pabst A.-S., Eid M.M., Thiele H., Nürnberg P., Budde B.S. (2023). Biallelic *MAD2L1BP* (p31comet) mutation is associated with mosaic aneuploidy and juvenile granulosa cell tumors. JCI Insight.

[8] Yost S., de Wolf B., Hanks S., Zachariou A., Marcozzi C., Clarke M., de Voer R.M., Etemad B., Uijttewaal E., Ramsay E. (2017). Biallelic *TRIP13* mutations predispose to Wilms tumor and chromosome missegregation. Nat. Genet..

[9] de Wolf E., Oghabian A., Akinyi M.V., Hanks S., Tromer E.C., van Hooff J.J.E., van Voorthuijsen L., van Rooijen L.E., Verbeeren J., Uijttewaal E.C.H. (2021). Chromosomal instability by mutations in the novel minor spliceosome component CENATAC. EMBO J..

[10] Snape K., Hanks S., Ruark E., Barros-Núñez P., Elliott A., Murray A., Lane A.H., Shannon N., Callier P., Chitayat D. (2011). Mutations in *CEP57* cause mosaic variegated aneuploidy syndrome. Nat. Genet..

[11] Guo J., He W.-B., Dai L., Tian F., Luo Z., Shen F., Tu M., Zheng Y., Zhao L., Tan C. (2024). Mosaic variegated aneuploidy syndrome with tetraploidy and predisposition to male infertility triggered by mutant *CEP192*. HGG Adv..

[12] Grange L.J., Reynolds J.J., Ullah F., Isidor B., Shearer R.F., Latypova X., Baxley R.M., Oliver A.W., Ganesh A., Cooke S.L. (2022). Pathogenic variants in *SLF2* and *SMC5* cause segmented chromosomes and mosaic variegated hyperploidy. Nat. Commun..

[13] Malumbres M., Villarroya-Beltri C. (2024). Mosaic variegated aneuploidy in development, ageing and cancer. Nat. Rev. Genet..

[14] Hirano T. (2016). Condensin-based chromosome organization from bacteria to vertebrates. Cell.

[15] Uhlmann F. (2016). SMC complexes: From DNA to chromosomes. Nat. Rev. Mol. Cell Biol..

[16] Yatskevich S., Rhodes J., Nasmyth K. (2019). Organization of chromosomal DNA by SMC complexes. Annu. Rev. Genet..

[17] De Piccoli G., Cortes-Ledesma F., Ira G., Torres-Rosell J., Uhle S., Farmer S., Hwang J.-Y., Machin F., Ceschia A., McAleenan A. (2006). Smc5-Smc6 mediate DNA double-strand-break repair. Nat. Cell Biol..

[18] Potts P.R., Porteus M.H., Yu H. (2006). Human SMC5/6 complex promotes sister chromatid homologous recombination. EMBO J..

[19] Zhao X., Blobel G. (2005). A SUMO ligase is part of a nuclear multiprotein complex. Proc. Natl. Acad. Sci. USA.

[20] Palecek J.J., Gruber S. (2015). Kite proteins: A superfamily of SMC/kleisin partners. Structure.

[21] Andrews E.A., Palecek J., Sergeant J., Taylor E., Lehmann A.R., Watts F.Z. (2005). Nse2, a component of the Smc5-6 complex, is a SUMO ligase. Mol. Cell Biol..

[22] Kolesar P., Stejskal K., Potesil D., Murray J.M., Palecek J.J. (2022). Role of Nse1 subunit as a ubiquitin ligase. Cells.

[23] Nasmyth K., Haering C.H. (2009). Cohesin: Its roles and mechanisms. Annu. Rev. Genet..

[24] Hirano T. (2012). Condensins: Universal organizers of chromosomes with diverse functions. Genes. Dev..

[25] Li H., Durbin R. (2009). Fast and accurate short read alignment with Burrows-Wheeler transform. Bioinformatics.

[26] Li H., Handsaker B., Wysoker A., Fennell T., Ruan J., Homer N., Marth G., Abecasis G. (2009). Durbin R; 1000 Genome Project Data Processing Subgroup. The Sequence Alignment/Map format and SAMtools. Bioinformatics.

[27] Ebbert M.T., Wadsworth M.E., Staley L.A., Hoyt K.L., Pickett B., Miller J., Duce J., Kauwe J.S.K., Ridge P.G. (2016). Evaluating the necessity of PCR duplicate removal from next-generation sequencing data and a comparison of approaches. BMC Bioinform..

[28] DePristo M.A., Banks E., Poplin R., Garimella K.V., Maguire J.R., Hartl C., Philippakis A.A., del Angel G., Rivas M.A., Hanna M. (2011). A framework for variation discovery and genotyping using next-generation DNA sequencing data. Nat. Genet..

[29] Yang Y., Zheng Y., Li W., Li L., Tu M., Zhao L., Mei H., Zhu G., Zhu Y. (2019). *SMAD6* is frequently mutated in nonsyndromic radioulnar synostosis. Genet. Med..

[30] Richards S., Aziz N., Bale S., Bick D., Das S., Gastier-Foster J., Grody W.W., Hegde M., Lyon E., Spector E. (2015). ACMG Laboratory Quality Assurance Committee. Standards and guidelines for the interpretation of sequence variants: A joint consensus recommendation of the American College of Medical Genetics and Genomics and the Association for Molecular Pathology. Genet. Med..

[31] Duan X., Yang Y., Chen Y.-H., Arenz J., Rangi G.K., Zhao X., Ye H. (2009). Architecture of the Smc5/6 complex of Saccharomyces cerevisiae reveals a unique interaction between the Nse5-6 subcomplex and the hinge regions of Smc5 and Smc6. J. Biol. Chem..

[32] Peng X.P., Zhao X. (2023). The multi-functional Smc5/6 complex in genome protection and disease. Nat. Struct. Mol. Biol..

[33] Li Q., Zhang J., Haluska C., Zhang X., Wang L., Liu G., Wang Z., Jin D., Cheng T., Wang H. (2024). Cryo-EM structures of Smc5/6 in multiple states reveal its assembly and functional mechanisms. Nat. Struct. Mol. Biol..

[34] Räschle M., Smeenk G., Hansen R.K., Temu T., Oka Y., Hein M.Y., Nagaraj N., Long D.T., Walter J.C., Hofmann K. (2015). Proteomics reveals dynamic assembly of repair complexes during bypass of DNA cross-links. Science.

[35] Schwertman P., Bekker-Jensen S., Mailand N. (2016). Regulation of DNA double-strand break repair by ubiquitin and ubiquitin-like modifiers. Nat. Rev. Mol. Cell Biol..

