# Supplementary material for: Recessive SMC5 Variants in a Family with Near-Tetraploidy/Mosaic Variegated Aneuploidy
Source: Diagnostics (Basel). 2025 Nov 27;15(23):3022. doi: 10.3390/diagnostics15233022 (PMC12691382; doi:10.3390/diagnostics15233022)
Supplement: Supplementary file 1 [file diagnostics-15-03022-s001.zip › diagnostics-3946524-supplementary.pdf]

Table S1. Demographic Clinical Characteristics of the Study Participants (as control)

Control-Individuals	Sex	Age (years)	Karyotype	Clinical Diagnosis
C1	M	5.2	46, XY	Short stature
C2	F	3.8	46, XX	Short stature
C3	M	7.1	46, XY	Hypospadias
C4	F	4.5	46, XX	Short stature
C5	F	9	46, XX,9qh+	Short stature
C6	M	6	46, XY	Hypospadias
C7	M	5.5	46, XY	Hypospadias
C8	F	6.8	46, XX	Short stature
C9	M	9.3	46, XY	Hypospadias
C10	M	10.7	46, XY	Short stature

Table S2. Results of the Initial Karyotype Analysis of the Proband with *SMC5* biallelic variants. **Note:** Due to excessive chromosome crowding in near-tetraploid cells, detailed pairwise analysis was not performed for Near-tetraploid cells.

Cell	Karyotype Analysis
1	Near-tetraploid
2	Near-tetraploid
3	Near-tetraploid
4	Near-tetraploid
5	Near-tetraploid
6	Near-tetraploid
7	Near-tetraploid
8	Near-tetraploid
9	Near-tetraploid
10	46,XY,21ps-
11	46,XY,21ps-
12	46,XY,del(18)(q22),21ps-
13	46,XY,21ps-
14	47,XY,+mar,21ps-
15	46,XY,21ps-
16	46,XY,21ps-
17	46,XY,21ps-
18	46,XY,21ps-

19	46,XY,21ps-
20	45,XY,-17,del(9)(q33),21ps-
21	46,XY,21ps-
22	46,XY,21ps-
23	46,XY,21ps-
24	46,XY,21ps-
25	46,XY,del(8)(q22),21ps-
26	46,XY,21ps-
27	42,XY,-4,-12,-13,-18,psu dic(3)t(12;3)(p12.2;p25),21ps-
28	46,XY,dup(5)(p11p15),21ps-
29	45,XY,-16,21ps-
30	46,XY,21ps-
31	46,XY,21ps-
32	46,XY,21ps-
33	46,XY,del(14)(p11.1),21ps-
34	46,XY,del(5)(q23),21ps-
35	46,XY,del(12)(q15),21ps-
36	46,XY,21ps-
37	43,XY,-2,-2,-7,-18,+mar,21ps-
38	46,XY,21ps-
39	46,XY,21ps-
40	46,XY,21ps-
41	42,XY,-7,-8,-14,-18,del(3)(q24);der(6)t(7;6)(q11;q16)del(11)(q14),+mar,21ps-
42	41,XY,-3,-5,-6,-12,-13,-14,-21,-21,+mar1,+mar2,+mar3,der(2)t(6;2)(q23;p25)
43	46,XY,-5,+mar,del(2)(q11),add(3)(q29),del(5)(p11),del(8)(q22),del(11)(q21),21ps-
44	46,XY,21ps-
45	46,XY,-21,+mar,der(10)t(21;10)(q11;qter),21ps-
46	46,XY,21ps-
47	46,XY,21ps-
48	41,XY,-6,-13,-19,-4,-4,21ps-
49	46,XY,del(11)(p13),21ps-
50	46,XY,21ps-
51	46,XY,-13,+mar,21ps-
52	46,XY,del(1)(p32)， 21ps-
53	46,XY,21ps-
54	46,XY,21ps-
55	46,XY,21ps-
56	47,XY,+mar,21ps-

57	46,XY,21ps-
58	46,XY,21ps-
59	46,XY,21ps-
60	46,XY,del(7)(q11),21ps-
61	46,XY,21ps-
62	46,XY,21ps-
63	46,XY,-21,+r,21ps-
64	46,XY,21ps-
65	46,XY,del(5)(q31)del(9)(q21),21ps-
66	47,XY,del(5)(q31),+mar,21ps-
67	46,XY,+mar1,+mar2,del(8)(q22),del(11)(p14),21ps-
68	48,XY,+mar1,+mar2,21ps-
69	46,XY,21ps-
70	47,XY,-16,+mar1,+mar2,21ps-
71	46,XY,21ps-

Figure S1: Workflow of variant filtering and analysis.

Focus on 10 known MVA pathogenic genes:
BUB1B, BUB1, MAD1L1, MAD2L1BP, TRIP13, CENATAC, CEP57, CEP192, SMC5, SLF2

↓

Retain rare coding variants:
frequency ≤ 2% in either gnomAD_Eas or gnomAD_All, version 4.1.0

↓

Remove variants:
1) synonymous changes not expected to affect the protein sequence; 2) absence of a predicted impact on splicing; and 3) presence of the variant in a homozygous state in the gnomAD_All cohort (version 4.1.0)

↓

The compound heterozygous variants in the SMC5 were the only ones that remained after filtering

Table S3. Results of the Second Karyotype Analysis of the Proband with *SMC5* biallelic variants

Cell	Karyotype Analysis
1	45,XY,-21,del(8)(p23.1)
2	46,XY,-2,-8,-11,+mar1,+mar2,+mar3,del(5)(p13),der(4)psu dic(4)t(2;4)(q21;q31),21ps-
3	47,XY,+7,del(8)(q21.3),21ps-
4	46,XY,21ps-
5	44,Y,-X,-6,-14,+15,+mar,t(3;6)(q25;p11),21ps-
6	39,XY,-1,-3,-15,-17,-18,-19,-20,del(6)(q25),21ps-
7	46,XY,21ps-
8	46,XY,21ps-
9	46,XY,21ps-
10	46,XY,21ps-
11	43,Y,-X,-3,-16,21ps-
12	46,XY,21ps-
13	46,XY,21ps-
14	46,XY,21ps-
15	46,XY,21ps-
16	46,XY,21ps-
17	45,XY,-9,der(1)t(9;1)(q22;q32),21ps-
18	46,XY,21ps-
19	44,XY,-7,-13,21ps-
20	46,XY,21ps-
21	46,XY,21ps-
22	46,XY,21ps-
23	46,XY,21ps-
24	45,XY,-22,21ps-
25	46,Y,-X,+19,21ps-
26	47,XY,+mar,21ps-
27	46,XY,21ps-
28	48,XY,+mar1,+mar2,21ps-
29	46,XY,21ps-
30	44,XY,-5,-6,-17,+mar,21ps-
31	46,XY,21ps-
32	40,XY,-2,-7,-14,-16,-17,-19,21ps-
33	45,XY,-13,21ps-
34	46,XY,-9,+20,21ps-
35	46,XY del(6)(q22.1),21ps-
36	46,XY,21ps-
37	50,XY,+4mar,21ps-
38	46,XY,21ps-
39	46,XY,21ps-

40	46,XY,21ps-
41	46,XY,21ps-
42	46,XY,21ps-
43	45,XY,-11,21ps-
44	46,XY,-20,+mar,21ps-
45	46,XY,21ps-
46	46,XY,21ps-
47	46,XY,21ps-
48	46,XY,21ps-
49	46,XY,21ps-
50	44,XY,-19,-22,+mar,21ps-
51	44,XY,-10,-13,21ps-
52	46,XY,21ps-
53	46,XY,21ps-
54	46,XY,del(10)(q22),21ps-
55	45,Y,-X,21ps-
56	46,XY,21ps-
57	46,XY,21ps-
58	46,XY,21ps-
59	46,XY,21ps-
60	46,XY,21ps-
61	45,XY,-19,21ps-
62	49,XY,-21,+mar1,+mar2,+mar3,+mar4
63	46,XY,21ps-
64	46,XY,21ps-
65	46,XY,21ps-
66	46,XY,21ps-
67	45,XY,-14,21ps-
68	48,XY,+2mar,21ps-
69	41,XY,-9,-10,-12,-14,-21,del(4)(q31)
70	42,XY,-14,-16,-20,-21
71	45,XY,del(9)(q21),21ps-
72	46,XY,21ps-
73	46,XY,21ps-
74	46,XY,21ps-
75	46,XY,-4,+12,21ps-
76	45,XY,-19,21ps-
77	45,XY,-12,21ps-
78	46,XY,21ps-
79	45,XY,-19,21ps-
80	45,X,-Y,der(3)t(12;3)(p11;pter) ,21ps-
81	46,XY,+11,-5,del(1)(p32),21ps-
82	46,XY,del(2)(q31),21ps-

83	46,XY,add(5)(p15.3),21ps-
84	46,XY,21ps-
85	45,XY,-12,21ps-
86	43,XY,-3,-15,-20,21ps-
87	46,XY,21ps-
88	45,XY,-2,21ps-
89	46,XY,del(8)(p12),del(9)(p22) ,21ps-
90	45,XY,-13,21ps-
91	43,XY,-5,-10,-18,21ps-
92	46,XY,21ps-
93	46,XY,21ps-
94	46,XY,-6,+mar,del(1)(p32),der(11)t(6;11)(p12;q23),21ps-
95	46,XY,-18,+mar,21ps-
96	46,XY,21ps-
97	46,XY,21ps-
98	46,XY,21ps-
99	44,XY,-13,-18,-22,+15,21ps-
100	46,XY,21ps-
101	46,XY,21ps-
102	45,XY,-4,-18,+mar,21ps-
103	46,XY,21ps-
104	46,XY,-20,-21,+mar,del(1)(p22)
105	45,XY,-22,del(17)(q24),21ps-
106	46,XY,21ps-
107	46,XY,21ps-
108	45,XY,-19,21ps-
109	51,XY,+2,+4mar,21ps-
110	46,XY,21ps-
111	46,XY,21ps-
112	46,XY,21ps-
113	46,XY,del(5)(q34),21ps-
114	46,XY,21ps-
115	46,XY,21ps-
116	45,XY,-13,del(3)(q21),21ps-
117	43,XY,-4,-5,-14,-16,+mar,del(1)(p31),21ps-
118	46,XY,21ps-
119	46,XY,21ps-
120	48,XY,-19,+mar1,+mar2,+mar3 ,21ps-
121	44,XY,-3,-20,21ps-
122	46,XY,del(6)(q23),21ps-
123	45,XY,-14,21ps-
124	46,XY,-12,+mar,21ps-
125	47,XY,+mar,21ps-

126	44,Y,-X,-12,21ps-
127	46,XY,21ps-
128	46,XY,-3,+mar,21ps-
129	46,XY,21ps-
130	46,XY,21ps-
131	46,XY,21ps-
132	46,XY,21ps-
133	47,XY,+mar,21ps-
134	46,XY,21ps-
135	46,XY,21ps-
136	46,XY,21ps-
137	46,XY,21ps-
138	46,XY,21ps-
139	46,XY,21ps-
140	46,XY,21ps-
141	40,XY,-6,-12,-18,del(11)(q23),del(17)(q21),21ps-
142	44,XY,-9,-13,-14,+11,21ps-
143	47,XY,-1,-12,+mar1,+mar2,+mar3,21ps-
144	46,XY,21ps-
145	46,XY,21ps-
146	47,XY,+mar,21ps-
147	46,XY,21ps-
148	46,XY,21ps-
149	46,XY,21ps-
150	46,XY,21ps-
151	46,XY,21ps-
152	46,XY,21ps-
153	45,XY,-19,21ps-
154	46,XY,21ps-
155	46,XY,21ps-
156	46,XY,-12,+mar,21ps-
157	45,XY,-5,+mar,21ps-
158	46,XY,21ps-
159	46,XY,21ps-
160	46,XY,21ps-
161	46,XY,del(8)(q22),21ps-
162	45,Y,-X,21ps-
163	46,XY,21ps-
164	46,XY,21ps-
165	46,XY,21ps-
166	46,XY,21ps-
167	46,XY,21ps-
168	46,XY,21ps-

169	47,XY,+mar,21ps-
170	44,XY,-8,-11,+der(8)t(11;8)(q13;q24),21ps-
171	46,XY,21ps-
172	44,XY,-2,-14,21ps-
173	47,XY,+mar,21ps-
174	46,XY,21ps-
175	47,XY,-17,-20,+mar1,+mar2,+mar3 ,21ps-
176	46,XY,21ps-
177	43,X,-Y,-4,-13,+mar,21ps-
178	44,XY,-17,-19,del(11)(q23),21ps-
179	46,XY,+13,-21
180	44,XY,-14,-16,del(2)(q33),del(9)(q22),21ps-
181	47,XY,+mar,21ps-
182	46,XY,21ps-
183	46,XY,t(8;10)(p23;p12),21ps-
184	46,XY,21ps-
185	46,XY,-5,-12,-14,-18,+mar1,+mar2,+mar3,+mar4,21ps-
186	46,XY,21ps-
187	46,XY,21ps-
188	46,XY,del(4)(p11),21ps-
189	45,XY,-16,21ps-
190	46,XY,21ps-
191	45,XY,-7,21ps-
192	45,XY,-3,21ps-
193	46,XY,21ps-
194	44,XY,-4,-13,21ps-
195	46,XY,21ps-
196	46,XY,21ps-
197	46,XY,21ps-
198	47,XY,+mar,21ps-
199	46,XY,21ps-
200	45,XY,-5,21ps-
201	46,XY,21ps-
202	46,XY,21ps-
203	46,XY,21ps-
204	46,XY,21ps-
205	45,XY,-20,21ps-
206	46,XY,21ps-
207	46,XY,21ps-
208	45,XY,-11,21ps-
209	46,XY,del(4)(q28),21ps-
210	46,XY,21ps-
211	46,XY,-15,+mar,21ps-

212	46,XY,21ps-
213	46,XY,21ps-
214	45,XY,-10,21ps-
215	46,XY,21ps-
216	42,Y,-X,-8,-17,-19,del(13)(q22),21ps-
217	46,XY,21ps-
218	47,XY,-6 +mar1,+mar2,21ps-
219	45,XY,-17,del(3)(q21),21ps-
220	46,XY,21ps-
221	46,XY,21ps-
222	47,XY,+mar,21ps-
223	46,XY,21ps-
224	47,XY,+mar,21ps-
225	46,XY,21ps-
226	43,XY,-6,-10,-18,-19,+der(10)t(10;18)(p15;p11),21ps-
227	46,XY,21ps-
228	46,XY,21ps-
229	46,XY,21ps-
230	45,XY,-3,-9,+der(3)t(3;9)(q28;q22),21ps-
231	46,XY,21ps-
232	46,XY,21ps-
233	47,XY,+mar,21ps-
234	45,XY,-13,21ps-
235	46,XY,21ps-
236	47,XY,+mar,21ps-
237	46,XY,21ps-
238	46,XY,-18,+20,21ps-
239	46,XY,-11,+mar,21ps-
240	46,XY,-4,+mar,21ps-
241	46,XY,21ps-
242	46,XY,21ps-
243	46,XY,-4,-5,-9,+mar1,+mar2,+mar3,21ps-
244	46,XY,21ps-
245	46,XY,chrb(12)(q13),add(5)(p15),add(9)(q34),21ps-
246	47,XY,+mar,21ps-
247	46,XY,21ps-
248	46,XY,21ps-
249	46,XY,21ps-
250	47,XY,-4,-16,+mar1,+mar2,+mar3,21ps-
251	46,XY,21ps-
252	45,XY,-16,21ps-
253	46,XY,del(7)(q21),21ps-
254	46,XY,21ps-

255	46,XY,21ps-
256	46,XY,21ps-
257	46,XY,21ps-
258	46,XY,21ps-
259	46,XY,21ps-
260	47,XY,+mar,21ps-
261	46,XY,21ps-
262	45,XY,-15,-21,+10,21ps-
263	46,XY,-20,+mar,21ps-
264	46,XY,21ps-
265	46,XY,del(6)(q22),21ps-
266	45,XY,-12,21ps-
267	46,XY,21ps-
268	46,XY,-17,+mar,21ps-
269	46,XY,21ps-
270	46,XY,21ps-
271	46,XY,21ps-
272	46,XY,21ps-
273	46,XY,21ps-
274	45,XY,-18,21ps-
275	46,XY,del(6)(q21),del(7)(p15),add(7)(p21),21ps-
276	47,XY,+21,21ps-
277	46,XY,21ps-
278	46,XY,21ps-
279	40,X,-Y,-3,-8,-10,-16,-21,-22,+r,21ps-
280	46,XY,21ps-
281	46,XY,-19,+mar,21ps-
282	46,XY,21ps-
283	46,XY,21ps-
284	46,XY,21ps-
285	45,XY,-18,-19,-20,+mar1,+mar2,21ps-
286	47,XY,+mar,21ps-
287	46,XY,21ps-
288	46,XY,21ps-
289	46,XY,21ps-
290	46,XY,21ps-
291	46,XY,21ps-
292	46,Y,-X,+mar,21ps-
293	46,XY,21ps-
294	44,XY,-15,-22,21ps-
295	44,XY,-12,-14,del(2)(p11.2),21ps-
296	46,XY,+3,-12,21ps-
297	44,XY,-8,-18,-21,+mar1,+mar2,21ps-

298	40,XY,-2-,4,-7,-12,-13,-21,t(1;2)(q44;q37.3),chtb(6)(q13),21ps-
299	46,XY,dup(14)(q22),21ps-
300	37,XY,-8,-9,-11,-13,-14,-14,-16,-17,-19,-22,+der(9)t(9;11)(q22;q23),21ps-
301	46,XY,21ps-
302	44,XY,-14,-19,21ps-
303	45,XY,-6,21ps-
304	43,XY,-4,-8,-17,chtb(9)(p22),21ps-
305	47,XY,-7,-8,-12,+5,+mar1,+mar2,+mar3,del(2)(p22),21ps-
306	43,XY,-6,-7,-14,-<br>18,+der(7)t(6;7)(p22;q11),del(9)(q22),del(11)(q23),add(11)(p15) ,21ps-
307	43,XY,+2,-5,-11,-15,-20,21ps-
308	46,XY,t(10;16)(p13;p13),21ps-
309	46,XY,-13,+mar,21ps-
310	43,XY,-2,-2,-18,+mar,del(6)(p22),21ps-
311	48,XY,+mar1,+mar2,21ps-
312	47,XXY,-5,-13,+mar1,+mar2,21ps-
313	47,XY,-18,+mar1,+mar2,21ps-
314	46,XY,del(4)(q33),21ps-
315	46,XY,-18,+mar,21ps-
316	46,XY,21ps-
317	46,XY,21ps-
318	47,XY,-2,+mar1,+mar2,21ps-
319	46,XY,21ps-
320	45,XY,-5,-8,-21ps-,+mar1,+mar2
321	45,XY,-16,21ps-
322	43,XY,-8,-15,-16,-18,-21,+mar1,+mar2,21ps-
323	43,XY,-3,-10,-20,del(11)(p12)
324	40,XY,-5,-7,-16,-16,-17,-21,-22,+mar,21ps-
325	46,XY,-2,+mar,21ps-
326	45,XY,-22,21ps-
327	46,XY,21ps-
328	44,X,-Y,-18,del(9)(p22),21ps-
329	45,XY,-20,21ps-
330	45,XY,-21ps-
331	44,XY,-10,-17,21ps-
332	46,XY,21ps-
333	46,XY,21ps-
334	45,XY,-20,21ps-
335	46,XY,21ps-
336	48,XY,+2mar,21ps-
337	46,XY,-6,-13,+mar1,+mar2,21ps-
338	43,XY,-2,-7,-12,-16,-20,-21ps-,+18,+mar1,+mar2
339	46,XY,-16,+mar,21ps-

340	45,XY,-12,-21ps-,+r
341	45,XY,-18,21ps-
342	46,XY,21ps-
343	44,XY,-2,-20,del(7)(p15)
344	45,XY,-20,21ps-
345	45,XY,-18,-19,+mar,add(7)(p21),21ps-
346	46,XY,21ps-
347	46,XY,21ps-
348	46,XY,21ps-
349	44,XY,-11,-20,21ps-
350	46,XY,21ps-
351	45,XY,-18,21ps-
352	44,XY,-18,-22,21ps-
353	44,XY,-18,-19,21ps-
354	46,XY,21ps-
355	42,XY,-10,-11,-17,-17,-18,+mar,21ps-
356	46,XY,21ps-
357	45,XY,-5,21ps-
358	41,XY,-13,-16,-19,-21ps-,-22
359	46,XY,21ps-
360	45,XY,-19,21ps-
361	46,XY,-21,+mar,21ps-
362	46,XY,del(2)(q36),21ps-
363	46,XY,21ps-
364	42,Y,-X,-9,-20,-22,21ps-
365	46,XY,del(6)(q25),21ps-
366	46,XY,-16,-20,+mar1,+mar2,21ps-
367	45,XY,-14,21ps-
368	45,XY,-6,-16,-18,+mar1,+mar2,21ps-
369	46,XY,21ps-
370	46,XY,21ps-
371	46,XY,-9,-15,+2mar,21ps-
372	46,XY,-8,-8,-12,-18,+mar1,+mar2,+mar3,+mar4,21ps-
373	50,XY,-4,-5,+mar1,+mar2,+mar3,+mar4,+mar5,+mar6,21ps-
374	45,XY,-11,21ps-
375	47,XY,-5,-18,+mar1,+mar2,+mar3,21ps-
376	46,XY,21ps-
377	44,XY,-17,-17,21ps-
378	46,XY,-6,+13,21ps-
379	45,XY,-15,21ps-
380	46,XY,21ps-
381	44,XY,-5,-9,-22,+mar,der(3)t(22;3)(q10;q25)
382	46,XY,-14,-16,-17,-18,+mar1,+mar2,+mar3,+mar4,21ps-

383	45,XY,-2,-8,+mar,21ps-
384	43,XY,-3,-5,-12,21ps-
385	46,XY,21ps-
386	38,XY,-4,-10,-12,-14,-15,-20,-21,-21
387	44,XY,-13,-20,21ps-
388	45,XY,rob(15;22)(q10;q10),21ps-
389	47,XY,+r,psu dic(5;7)(q22;q11)
390	46,XY,-16,+11,21ps-
391	46,XY,21ps-
392	46,XY,21ps-
393	46,XY,21ps-
394	45,XY,-21,del(5)(q31),del(11)(q23),21ps-
395	47,XY,-15,+mar1,+mar2,21ps-
396	46,XY,21ps-
397	45,XY,-18,21ps-
398	46,XY,21ps-
399	46,XY,21ps-
400	45,X,-Y,21ps-
401	45,XY,-7,-18,21ps-
402	45,XY,-20,21ps-
403	46,XY,21ps-
404	45,XY,-21ps-
405	46,XY,21ps-
406	43,X,-Y,-19,-22,21ps-
407	45,XY,-19,21ps-
408	46,XY,21ps-
409	45,XY,-17,21ps-
410	46,XY,del(8)(q11),21ps-
411	44,XY,-9,-20,21ps-
412	43,Y,-X,-2,-13,21ps-
413	45,XY,-8,21ps-
414	46,XY,21ps-
415	46,XY,-16,-19,-21ps-,+mar1,+mar2,+mar3
416	45,XY,-14,-18,+mar,21ps-
417	46,XY,21ps-
418	46,XY,-19,+mar,21ps-
419	45,X,-Y,del(1)(q42),21ps-
420	46,XY,del(5)(p13),21ps-
421	45,XY,-18,21ps-
	Near-tetraploid cell×43

Table S4. Results of Karyotype Analysis of cases with *CEP192* biallelic variants (Excluding Tetraploid Cells, 72 Abnormal Cells Observed)

**Cell**	**Karyotype Analysis**	**Count**
/	**case 1**	
1-7	92,XXXX (*)	7
8	47,XXX	1
9	47,XX,+mar	1
10	46,XX,del(3)(q27)	1
11	46,XX,del(6)(p21)	1
12	48,XX,+8,+18	1
13	45,XX,-13	1
14	45,XX,-14	1
15	47,XX,+15	1
16	45,XX,-16	1
17-18	45,XX,-18	2
19	47,XX,+18, del (6)(q22)	1
20	47,XX,+20	1
21	46,XX,del(20)(q13)	1
22	46,XX,del(21)(q22)	1
23	45,XX,-22	1
24-81	46,XX	58
110	92, XXXX	29
111	45,X0	1
112-113	47,XX,+mar	2
114	48,XX,+1,+11	1
115	45,XX,-5	1
116	46,XX,del(5)(p15)	1
117	45,XX,-6	1
118	45,XX,-7	1
119	45, XX,-8, t(2;11)(p10; q12)	1
120	48,XX,+8,+10	1
121	46,XX, del(7)(q32)	1
122	47,XX,-8, +mar1, +mar2	1
123	45,XX,-9	1
124	48,XX,+11,+22	1
125	46,XX,del(16)(q21)	1
126-127	45,XX,-12	2
128	45,XX,-14	1
129	45,XX, -16	1
130	47, XX,+16	1
131-134	47,XX,+18	4
135-136	45,XX,-18	2
137	46,XX,del(18)(q22)	1

138	46, XXX,-19	1
139	47, XX,+19	1
140	45,XX,-20	1
141-142	45,XX,-22	2
143	45, XX, -22, del (16)(q22)	1
144-202	46, XX	59
/	**case 2**	
203-223	92,XXYY	21
224	46,XY,del(2)(p23), del(16)(q23)	1
225-226	45,XY,-4	2
227	46, XY, del(5)(p15)	1
228	45, XY, -6	1
229	46, XY, -13, +18	1
230-231	46, XY, del (16)(q23)	2
232-237	47,XY,+18	6
238	46,XY,+18, -21	1
239-240	46, X, -Y, +18	2
241-242	45, XY, -19	2
243-246	45, XY, -21	4
247-289	46, XY	43
